# Impact of Delayed Trauma Unit Admission on Mortality and Disability in Traumatic Brain Injury Patients

**DOI:** 10.3390/ijerph22101566

**Published:** 2025-10-15

**Authors:** Julio Quispe-Alcocer, Antonio Biroli, Fabricio González-Andrade

**Affiliations:** 1 Health Sciences College, School of Medical Specialties, Universidad San Francisco de Quito USFQ, Diego de Robles Street s/n and Pampite, Quito 170901, Ecuador; dr.julioquispe@gmail.com; 2 Service of Neurosurgery, Hospital Eugenio Espejo, Av. Gran Colombia, Quito 170136, Ecuador; antoniobiroli@yahoo.com; 3 Facultad de Ciencias de la Salud y Bienestar, Dirección de Posgrados en Salud, Universidad Tecnológica Indoamérica, Calle Machala y Sabanilla, Quito 170301, Ecuador

**Keywords:** mortality, disability, clinical results, Brain trauma injury, arrival time at the hospital, Ecuador

## Abstract

Traumatic brain injury (TBI) remains a critical public health issue worldwide, with significant morbidity, mortality, and long-term disability. Timely transfer to a specialized trauma unit is crucial to improving outcomes, yet in resource-limited settings, delays often exceed recommended time frames. This study evaluates the impact of arrival time on mortality, disability, and clinical outcomes in Ecuadorian patients with TBI. A cross-sectional and observational study was conducted, analyzing 383 adult patients diagnosed with TBI. Patients were categorized into two cohorts: those who arrived at a specialized trauma unit within five hours post-injury and those who arrived between five and 24 h. Demographic, clinical, and radiological characteristics were analyzed, including Glasgow Coma Scale (GCS), Injury Severity Score (ISS), Marshall Scale classification, and presence of subarachnoid hemorrhage (SAH). Logistic regression models were used to identify predictors of mortality and disability. Longer transfer times were associated with increased mortality (3.34 times higher for ≥5 h, *p* < 0.05) and disability (2.92 times higher for ≥5 h, *p* < 0.05). Patients with Marshall Diffuse Injury III and IV had an 8.80- and 9.05-fold increased risk of mortality, respectively. SAH was an independent predictor of mortality (4.53 times higher), and GCS between 9–13 increased the likelihood of death by 6.49 times. Delayed transfers were associated with lower GCS at admission, longer ICU stays, and increased surgical complications. Although some survivors experienced improvement over time, disability in TBI can persist for many years or even lifelong, underscoring the burden of delayed trauma care. Despite delays, overall survival remained higher than reported in high-income countries, suggesting compensatory factors in hospital-based management. Delayed hospital arrival in TBI patients significantly increases mortality and disability. Early transfer within five hours is essential to reduce secondary brain injury and improve functional outcomes. Findings suggest that in resource-limited settings, optimizing pre-hospital care and transport efficiency is crucial to minimizing long-term disability.

## 1. Introduction

Traumatic brain injury (TBI) is a major global public health concern, with rising morbidity and mortality rates that impose significant healthcare costs. The economic burden extends beyond hospitalization, as long-term care and rehabilitation remain essential components of patient management [1]. According to the World Health Organization (WHO), TBI encompasses mild, moderate, and severe injuries, all of which can contribute to long-term neurological and psychosocial impairment [2]. The global incidence of TBI is estimated at 939 cases per 100,000 people, affecting approximately 69 million individuals annually [2]. Mortality rates are disproportionately higher in rural areas and in low- and middle-income countries compared to urban settings and high-income nations [3]. The most affected demographic is economically active individuals under the age of 30 [4].

Since the 1990s, prompt transfer of trauma victims to medical facilities has been recognized as a critical determinant of survival. The concept of the “golden hour”—emphasizing stabilization and initial treatment within the first 60 min—has evolved into the broader framework of the “golden period in trauma,” which advocates for definitive treatment within 90 min post-injury. This urgency is underscored by data indicating that mortality increases by 1% for every 3-min delay in polytrauma cases [5]. Optimal transport time for TBI patients to a specialized trauma center is estimated between 90 and 120 min, allowing for initial stabilization at primary care facilities [6]. In developed countries, inter-hospital transfer to a trauma unit typically takes between 2.5 and 4 h [7]. However, in developing nations, where healthcare infrastructure, economic constraints, geographical barriers, and legislative gaps limit pre-hospital transport access, the World Health Organization recommends transfer to a specialized trauma unit within 4 to 6 h [8].

TBI management is guided by the injury mechanism, and early transportation to trauma units enables a comprehensive approach, particularly for patients with polytrauma and severe TBI. Timely therapeutic interventions, such as securing the airway, thoracotomy, hemorrhagic shock management, intravenous medication administration, and orthopedic stabilization, have demonstrated a reduction in complications in up to 37% of polytrauma patients [9]. Additionally, early specialized care mitigates secondary complications, including aspiration pneumonia, anemia, hypothermia, infection, electrolyte imbalances, and seizures. Prompt neurosurgical intervention also shortens intensive care unit (ICU) and hospital stays, facilitates early discharge, and reduces overall complications [10].

Specialized trauma centers in developed nations emphasize the importance of arrival time relative to distance, defining urban areas as regions within 90 km or reachable within 60 min, while rural areas exceed these limits. Mortality rates among polytrauma and severe TBI patients differ significantly by setting, with urban mortality at 64% and rural mortality at 90% [11]. Trauma severity is compounded by time constraints; hemodynamically unstable patients with penetrating injuries experience a threefold increase in mortality for every 10-min delay in definitive surgical intervention [12].

TBI is a dynamic process, where the primary injury occurs immediately upon impact, while secondary injuries—such as cerebral edema and infarction—develop due to hypoxia, hypovolemia, hypothermia, or seizures [13]. Long-term disability following TBI is not limited to three years but may persist for decades or throughout the patient’s lifetime, affecting neurological, psychiatric, respiratory, and gastrointestinal functions [14]. Survivors often require lifelong care for persistent vegetative states, tracheostomy or gastrostomy dependence, motor deficits, cognitive impairments, and psychiatric disorders. Timely transfer to a specialized trauma center optimizes secondary injury management, reducing disability rates in TBI survivors [15].

This study was conducted in Quito, Ecuador, within two national reference hospitals. Ecuador’s healthcare system is segmented into public, social security, and private sectors, with universal access to emergency care guaranteed by law, though resource constraints and uneven insurance coverage often delay treatment. Emergency departments follow standardized protocols for TBI management, including the Glasgow Coma Scale (GCS), computed tomography (CT) of the brain, and initial airway and hemodynamic stabilization.

This study aims to analyze the relationship between arrival time at trauma units and clinical outcomes, including mortality and disability, in adult Ecuadorian patients with TBI.

## 2. Methods

**Study design:** This was an epidemiological, observational, cross-sectional, and multicenter study.

**Settings:** Hospital Eugenio Espejo and Hospital Carlos Andrade Marín from Quito, Ecuador. Although these hospitals receive patients transferred from rural and regional facilities across the country, the study population reflects outcomes in an urban tertiary-care setting.

**Participants:** The study included adult Ecuadorian patients (≥18 years of age) of both sexes and all ethnic groups, admitted with a diagnosis of TBI. Inclusion criteria were: (i) complete clinical records available at admission, (ii) brain imaging with CT or MRI, (iii) requirement for hospital admission (observation, general ward, or ICU), and (iv) informed consent provided by the patient or a legally authorized representative. Patients without complete medical records or without consent were excluded.

**Variables:** The following variables were collected from standardized data sheets for each eligible patient: sex, age, place of origin (city coded), mechanism of trauma (traffic accident: vehicle or motorcycle, driver or passenger; penetrating trauma: firearm or stabbing weapon; physical violence; fall <1.5 m or ≥1.5 m), personnel providing first aid at the scene (trained or untrained), type of transport to hospital (ambulance with physician, ambulance with paramedic, police car, taxi, private vehicle), GCS, transfer time (interval between trauma and arrival at trauma unit), sedation during transport, polytrauma defined as ISS > 16, motor response, pupillary response, presence of hypoxia, hypotension, hyperglycemia, and acute anemia. Radiological variables included Marshall CT classification (Diffuse I, II, III, IV, evacuated mass, non-evacuated mass) and presence of SAH. Treatment variables included surgical intervention and time to surgery, reinterventions, surgical complications, ICU admission, length of ICU stay, and degree of disability according to the Glasgow Outcome Scale (GOS) at hospital discharge. Disability was defined as GOS 2 (vegetative state) or GOS 3 (severe disability).

**Data sources and collection:** A coded database was designed, with unique identifiers for each hospital. Data were collected from medical records, pre-hospital transport sheets, and radiological reports. Trauma mechanisms and transfer details were verified from pre-hospital documentation. All clinical, laboratory, and imaging variables were standardized according to validated scales.

**Data measurement:** The researchers processed the data in an electronic matrix with the information of all the participants for subsequent calculation in statistical software.

**Biases avoided:** the data collected were stratified by clinical, radiological, and laboratory scales, with their respective validation, and variables with dichotomous options, avoiding subjective data at all costs.

**Study size:** *n* = 383

**Statistical techniques:** All analyses were performed using IBM SPSS version 25. Descriptive statistics were generated for demographic, clinical, and radiological variables. Categorical variables were expressed as absolute and relative frequencies; continuous variables were summarized using means and standard deviations or medians and interquartile ranges as appropriate. Inferential analysis included chi-square tests for categorical variables and Mann–Whitney tests for continuous variables. Variables significant in bivariate analyses were included in multivariate logistic regression models to identify predictors of disability and mortality. Odds ratios (OR) with 95% confidence intervals (CI) were calculated. Statistical significance was set at *p* < 0.05. Variables were considered risk factors if the lower CI limit was >1 and protective factors if the upper limit was <1.

**Ethical approval:** The institutional ethics committee approved the study protocol. At hospital admission, patients who were conscious and had capacity signed informed consent for the collection and research use of their medical history data. When patients lacked capacity (e.g., vegetative state) or were otherwise unable to sign, consent was obtained from a legally authorized representative/surrogate. For patients who subsequently died, consent had been secured at admission or from the representative during hospitalization. All information was anonymized prior to analysis, kept strictly confidential, and securely retained by the research group. Institutional Review Board Statement: The study was conducted in accordance with the Declaration of Helsinki and approved by the Ethics Committee on Human Re-search of the Universidad San Francisco de Quito (CEISH-USFQ), with the report IE02-EX103.2020-CEISH-USFQ.

## 3. Results

A total of 383 patients with a diagnosis of traumatic brain injury (TBI) were included in the analysis.

**Demographic and clinical characteristics**

Table 1 summarizes patient characteristics at admission according to time of arrival. The mean age was 56.8 years (SD ± 21.9), with a predominance of males (80.2%). The most frequent mechanisms of injury were falls from a height < 1.5 m (27.8%), traffic accidents involving drivers (22.9%), falls ≥1.5 m (18.7%), and physical violence (11.6%). Initial care was provided by physicians in 60.6% of cases. Polytrauma (ISS > 16) was present in 13.1% of patients, the mean ISS was 8.1, and the mean GCS score at admission was 10. Sedation during transport was required in 29.0% of patients.

Of the total, 28.2% arrived at the trauma unit within five hours, while 71.8% arrived ≥5 h post-injury. Patients who arrived within five hours had better outcomes at discharge: 46.3% achieved good recovery (GOS 5) compared with 28.0% of patients in the ≥5-h group (*p* = 0.012). When disability and mortality outcomes were combined, the proportions were 53.7% in the <5-h group versus 72.0% in the ≥5-h group, highlighting the adverse impact of delayed admission.

**Radiological characteristics**

Table 2 shows prognostic radiological findings according to arrival time. Marshall CT classification differed significantly between groups (*p* = 0.049). Among patients arriving within five hours, 18.5% were classified as Diffuse Injury I compared with 10.2% in the ≥5-h group. Conversely, Diffuse Injury II was more frequent in delayed patients (45.1%) compared with early arrivals (37.0%). Rates of subarachnoid hemorrhage (SAH) were similar between groups (73.2% vs. 74.2%, *p* = 0.836).

**Treatment and hospital course**

Table 3 summarizes treatment and hospital course. Surgical treatment was performed in 70.5% of cases, with no significant differences between groups. However, time from trauma to surgery was markedly shorter for early arrivals (mean 5.6 h) compared with delayed arrivals (mean 10.0 h, *p* < 0.001). ICU admission rates did not differ significantly (64.8% vs. 72.4%, *p* = 0.146). ICU stay ≥7 days was slightly more common among patients arriving late (61.4% vs. 55.7%), although not statistically significant. Post-surgical complications (30.6% vs. 25.1%, *p* = 0.276) and surgical reintervention rates (11.1% vs. 9.8%, *p* = 0.707) were also comparable.

**Predictors of disability**

Table 4 presents the multivariate logistic regression model for disability (GOS 2–3). The model explained 50% of variance (correlation coefficient = 0.50) and correctly classified 81.4% of cases. Arrival ≥5 h was associated with a 2.92-fold increased risk of disability (*p* = 0.003). Age > 62 years increased disability risk by 2.65 times (*p* = 0.002). Marshall Diffuse Injury III was the strongest predictor, conferring an 11.55-fold increased likelihood of disability (*p* = 0.001). GCS scores between 3–8 and 9–13 was associated with 5.07-fold (*p* < 0.001) and 3.77-fold (*p* = 0.029) increased risks of disability, respectively.

**Predictors of mortality**

Table 5 summarizes the multivariate model for mortality. The model explained 64% of variance (correlation coefficient = 0.64) with a predictive accuracy of 78%. Arrival ≥5 h increased the odds of death by 3.34 times (*p* = 0.012). Marshall Diffuse Injury III and IV were associated with 8.80-fold (*p* = 0.010) and 9.05-fold (*p* = 0.012) increased risks of mortality, respectively. The presence of SAH increased mortality by 4.53 times (*p* = 0.006). GCS scores between 9–13 was independently associated with higher mortality (OR = 6.49, *p* = 0.007).

## 4. Discussion

This study highlights the critical impact of delayed transfer to specialized trauma units on clinical outcomes in patients with traumatic brain injury (TBI). Patients were stratified based on arrival time, with one cohort transferred within five hours and the other arriving between five- and 24-h post-trauma. The findings demonstrate a direct association between prolonged transfer times and higher complication rates, longer intensive care unit (ICU) stays, delayed surgical interventions, and increased mortality and disability. Prognostic factors such as hypotension, anemia, polytrauma (ISS ≥ 16), Marshall classification higher than grade I, and Glasgow Coma Scale (GCS) scores below nine were more prevalent among late arrivals and were strongly correlated with unfavorable outcomes. Mortality exhibited a linear increase with longer transfer times, and the severity of disability in survivors also escalated, emphasizing the importance of timely access to specialized trauma care.

**Impact of early intervention in TBI management**

A systematic and timely approach to airway management, ventilation, and hemodynamic stabilization remains essential in trauma care. In TBI, these parameters directly influence the risk of secondary brain injury and the associated inflammatory cascade. Pre-hospital stabilization and rapid transfer to specialized trauma units are therefore critical to reduce hypoxemia and hypotension, both of which are strongly linked to worse outcomes [16,17]. Our findings support this: patients arriving later than five hours had nearly threefold higher mortality and disability, particularly when resuscitation was inadequate. The availability of trained personnel during transport was also associated with improved survival and fewer complications, reinforcing the importance of structured pre-hospital systems [18].

However, in low- and middle-income countries (LMICs) such as Ecuador, barriers including geographic distance, economic constraints, and limited emergency medical training contribute to prolonged transfer times. National data show that specialized trauma and neurosurgical centers are concentrated in urban areas, with limited capacity in rural regions. This leads to systemic delays in definitive care, a finding echoed in other LMIC settings [19,20].

**Neurological and radiological assessment in prognosis**

Early neurological and radiological assessment is vital to TBI management. Indicators such as altered pupillary responses, GCS < 9, and abnormal motor responses should immediately trigger transfer to a trauma unit and consideration for neurosurgical intervention [18,19]. Radiological findings also play a decisive role in prognosis: the presence of subarachnoid hemorrhage (SAH) and higher Marshall classifications are consistently linked to worse outcomes [20,21]. In our cohort, SAH independently increased the risk of death by 4.5 times, while Marshall Diffuse Injury III and IV were among the strongest predictors of mortality and disability. These results reinforce that prompt CT imaging, coupled with rapid surgical or critical care intervention, is crucial to prevent deterioration.

**Influence of trauma mechanism and polytrauma**

The severity of TBI outcomes is strongly influenced by the kinetic energy of the trauma. High-energy mechanisms, especially when associated with polytrauma (ISS ≥ 16), worsen prognosis by increasing mortality and limiting functional recovery [22]. Polytrauma amplifies mortality risk by more than 20-fold in hypotensive patients and remains an independent predictor of death regardless of transfer time [23,24]. In our study, polytrauma patients demonstrated markedly worse outcomes, underscoring the need for rapid multidisciplinary intervention in specialized centers. Evidence indicates that early correction of hypoxia, hypotension, hypothermia, and metabolic acidosis significantly improves survival in this subgroup [25,26,27].

**Context of the Ecuadorian health system**

Unlike most prior studies conducted in high-income countries, our analysis was situated in Ecuador. The Ecuadorian health system is segmented into three sectors: (i) the public Ministry of Health, (ii) social security institutions, and (iii) private care. Although universal access to emergency services is guaranteed by law, differences in insurance coverage and regional disparities affect the speed and quality of care. In practice, patients often face delays in pre-hospital transport and inter-hospital transfer due to limited ambulance networks and uneven distribution of neurosurgical services.

Emergency departments in Ecuador follow standardized protocols for TBI: initial evaluation using the Glasgow Coma Scale, airway stabilization, hemodynamic support, and urgent neuroimaging (CT scan) when available. Patients with abnormal findings are referred to neurosurgery for timely intervention. However, shortages of trained personnel, delays in diagnostic imaging, and resource constraints can compromise adherence to these guidelines, particularly outside major cities. These systemic challenges partly explain the delayed admissions observed in our cohort, while also contextualizing the relatively higher survival compared with some international cohorts.

**Economic and healthcare implications**

Early and targeted TBI management reduces not only complications but also healthcare costs by minimizing ICU stay and long-term disability [28]. In our cohort, late-arriving patients required longer hospitalization, had more complications, and often needed surgical reinterventions. While mortality in Ecuadorian patients was lower than anticipated, the functional burden of disability was significant, consistent with the observation that survivors frequently experience lifelong impairments. Previous reports that disability may last “up to three years” underestimate the burden; evidence indicates that many survivors remain disabled for decades or permanently, requiring long-term rehabilitation, assisted living, or continuous medical support [14].

**Reconsidering the “Golden Hour” and the “Golden Day”**

The principle of the “golden hour” has traditionally emphasized treatment within 60 min, but more recent evidence suggests that outcomes remain favorable if patients reach specialized care within four hours [29]. For patients with severe trauma, penetrating injuries, hypotension, or severe TBI, delays beyond this window are consistently associated with excess mortality [30]. In LMICs, however, systemic healthcare limitations often extend transfer times to 6–24 h. In our cohort, only 28% of patients reached a trauma unit within five hours, reflecting significant gaps in the emergency care system.

Interestingly, despite these delays, survival rates in our study exceeded those reported in some high-resource settings such as the United States and the United Kingdom [31]. We propose the concept of a “golden day” in the context of LMIC trauma systems: while early transfer remains the goal, survival may still be achieved beyond the conventional four- to six-hour window, though at the cost of substantially increased disability. This observation aligns with reports from other developing nations [32,33] and highlights the urgent need for structural reforms in pre-hospital and referral systems.

## 5. Conclusions

This study demonstrates that delayed admission to specialized trauma units significantly worsens outcomes in patients with traumatic brain injury (TBI). Arrival ≥5 h after injury was associated with a 3.34-fold higher probability of death and a 2.92-fold higher risk of disability. Marshall Diffuse Injury III and IV were among the strongest prognostic indicators, increasing mortality 8.80- and 9.05-fold, respectively. The presence of subarachnoid hemorrhage increased mortality 4.53-fold, while a GCS between 9–13 conferred a 6.49-fold higher probability of death. In predicting disability, advanced age (>62 years), Marshall Diffuse Injury III, and GCS < 13 was independently associated with poor outcomes.

Our findings confirm that hospital arrival should not exceed five hours post-trauma, as delays substantially increase both mortality and long-term disability. Importantly, disability following TBI is not restricted to the first three years but may persist for decades or throughout the patient’s lifetime, imposing a heavy burden on patients, families, and healthcare systems.

Within the Ecuadorian context, where tertiary trauma centers are concentrated in urban areas and pre-hospital resources are limited, many patients arrive later than recommended. Nevertheless, survival rates in this cohort were higher than expected compared to some high-income settings. This suggests that in resource-limited systems, aggressive hospital-based management may compensate for delayed transfer, though at the cost of greater disability.

These findings underscore the urgency of strengthening pre-hospital emergency systems, improving transport networks, and decentralizing trauma care in low- and middle-income countries. Early referral to trauma units remains essential, and the concept of a “golden day” may offer a more realistic framework for TBI management in LMICs, where systemic barriers often preclude transfer within the conventional “golden hour.”

## Figures and Tables

**Table 1:** Distribution of patients with TBI by arrival time at the hospital, according to clinical characteristics of admission.

Clinical Findings at Admission	General	Time of Arrival (Hours)		*p*-Value
		<5	≥5	
Age (mean (SD)) ^1/^	56.81 (21.95)	54.05 (23.00)	57.89 (21.47)	0.172
Sex (n (%)) ^2/^				
Male	307 (80.16)	88 (81.48)	219 (79.64)	0.684
Female	76 (19.84)	20 (18.52)	56 (20.36)	
Trauma mechanism (*n* (%)) ^2/^				
Minor fall than 1.5 m	101 (27.82)	26 (24.53)	75 (29.18)	0.502
Major fall than 1.5 m	68 (18.73)	21 (19.81)	47 (18.29)	
Road traffic accident: driver	83 (22.87)	26 (24.53)	57 (22.18)	
Road traffic accident: passenger	41 (11.29)	16 (15.09)	25 (9.73)	
Motorcycle	25 (6.89)	8 (7.55)	17 (6.61)	
Run over	3 (0.83)	0 (0)	3 (1.17)	
Physical violence	42 (11.57)	9 (8.49)	33 (12.84)	
First care (*n* (%)) ^2/^				
Medical	232 (60.57)	58 (53.7)	174 (63.3)	0.085
No Medical	151 (39.43)	50 (46.3)	101 (36.7)	
GOS (media (SD) ^1/^	12 (8–14)	12 (8–14)	12 (8–14)	0.691
Sedation (*n* (%)) ^2/^	111 (28.98)	33 (30.56)	78 (28.36)	0.671
Polytrauma (*n* (%)) ^2/^	50 (13.05)	11 (10.19)	39 (14.18)	0.296
ISS (media (SD) ^1/^	4 (1–16)	4 (1–16)	4 (1–16)	0.538
Hospital discharge condition **GOS (*n* (%))				
5 = good condition	127 (33.16)	50 (46.3) ^a^	77 (28) ^a^	0.012 *
4 = moderate disability	59 (15.4)	12 (11.11)	47 (17.09)	
3 = severe disability	63 (16.45)	14 (12.96)	49 (17.82)	
2 = Vegetative status	34 (8.88)	6 (5.56)	28 (10.18)	
1 = Death	100 (26.11)	26 (24.07)	74 (26.91)	

Nota: SD = standard deviation; ^1/^ = based on Mann–Whitney test. ^2/^ = based on the chi-square statistical homogeneity test. * significant differences in the proportions. equal superscripts indicate categories that differ **GOS. 5 = Good condition (minor deficit. leads an everyday life). 4 = Moderate disability (moderate deficit. but is independent). 3 = Severe disability (severe deficit with dependence on others for basic activities of daily living). 2 = Vegetative status (alive but not conscious. no verbal response. may open eyes). 1 = death (deceased attributable to TBI).

**Table 2:** Distribution of patients with TBI by arrival time at the hospital according to prognostic and radiological parameters.

Prognostic and Radiological Parameters	Arrival Time (Hours)		*p*-Value
	<5	≥5	
Motor response (*n* (%))			
Pathological extension	3 (2.88)	21 (7.66)	0.197
Pathological flexion	11 (10.58)	16 (5.84)	
Normal flexion	29 (27.88)	89 (32.48)	
Locate	21 (20.19)	55 (20.07)	
Obey	40 (38.46)	93 (33.94)	
Pupillary response (*n* (%))			
Reactive	88 (81.48)	240 (87.27)	0.320
One-sided reactivity	17 (15.74)	31 (11.27)	
No reactivity	3 (2.78)	4 (1.45)	
Hypoxia (*n* (%))	27 (25)	66 (24)	0.837
Hypotension (*n* (%))	21 (19.44)	56 (20.36)	0.840
Hyperglycemia (*n* (%))	91 (84.3)	247 (89.8)	0.128
Acute anemia (*n* (%))	18 (16.67)	35 (12.73)	0.315
Marshall scale (*n* (%))			
Diffuse I	20 (18.52) ^a^	28 (10.18) ^a^	0.049 *
Diffuse II	40 (37.04) ^a^	124 (45.09) ^a^	
Diffuse III	32 (29.63)	96 (34.91)	
Diffuse IV	16 (14.81)	27 (9.82)	
Subarachnoid hemorrhage (*n* (%))	79 (73.15)	204 (74.18)	0.836
Evacuate lesion (*n* (%))	16 (14.81)	27 (9.82)	0.163

Note: based on the chi-square test for statistical homogeneity; * significant differences in proportions. Equal superscripts indicate categories that differ.

**Table 3:** Distribution of patients with TBI by arrival time at the hospital according to treatment received.

Treatment	Arrival Time (Hours)		*p*-Value
	<5	≥5	
Surgical treatment (*n* (%)) ^1/^	74 (68.52)	200 (72.73)	0.411
Trauma time to surgery (mean (SD)) ^2/^	5.66 (0.67)	10 (4.07)	0.000 *
Admission to ICU (*n* (%)) ^1/^	70 (64.81)	199 (72.36)	0.146
ICU stay (*n* (%)) ^1/^			
<7 days	31 (44.29)	78 (38.61)	0.780
≥7 days	39 (55.71)	124 (61.39)	
Post-surgical complications (*n* (%)) ^1/^	33 (30.56)	69 (25.09)	0.276
Surgical reintervention (*n* (%)) ^1/^	12 (11.11)	27 (9.82)	0.707

Note: ^1/^ based on the chi-square test of statistical homogeneity; ^2/^ based on Mann–Whitney. * significant differences in means.

**Table 4:** Multivariate relationship to predict disability in patients with TBI.

Variables	B	*p*-Value	OR	CI-OR 95%	
				Lower	Upper
Arrival time (≥5 h late)	1.07	0.003 *	2.92 **	1.46	5.86
Age > 62 years	0.98	0.002 *	2.65 **	1.43	4.94
Male sex	0.61	0.115	1.84	0.86	3.94
Marshall					
Diffuse II	−0.04	0.930	0.96	0.35	2.59
Diffuse III	2.45	0.001 *	11.55 *	2.81	47.41
Diffuse IV	−0.04	0.964	0.96	0.18	5.25
Subarachnoid hemorrhage (yes)	0.20	0.562	1.22	0.63	2.35
Glasgow					
3 to 8	1.62	0.000 *	5.07 *	2.31	11.12
9–13	1.33	0.029 *	3.77 *	1.15	12.36

Note: * significant variable *p*-value < 0.05. ** OR = significant odds ratio; based on logistic regression.

**Table 5:** Multivariate relationship to predict mortality in patients with TBI.

Variables	B	*p*-Value	OR	CI-OR 95%	
				Lower	Upper
Arrival time (≥5 h late)	1.21	0.012 *	3.34 **	1.31	8.55
Age > 62 years	0.13	0.753	1.14	0.50	2.59
Male ex	0.40	0.413	1.49	0.57	3.87
Marshall					
Diffuse II	−0.48	0.487	0.62	0.16	2.39
Diffuse III	2.17	0.010 *	8.80 **	1.67	46.33
Diffuse IV	2.20	0.012 *	9.05 **	1.62	50.47
Subarachnoid hemorrhage (yes)	1.51	0.006*	4.53 **	1.53	13.41
Glasgow					
3–8	0.86	0.135	2.36	0.77	7.27
9–13	1.87	0.007 *	6.49 **	1.66	25.41

Note: * significant variable *p*-value < 0.05. ** OR = significant odds ratio; based on logistic regression.

## Data Availability

The data that supports this manuscript is and will remain stored by the authors; access to it will be made upon request.

## Abbreviations

The following abbreviations are used in this manuscript:

CT	computed axial tomography
GCS	Glasgow Coma Scale
GOS	Glasgow Outcome Scale
ICU	intensive care unit
ISS	Injury Severity Score
MRI	magnetic resonance imaging
SAH	subarachnoid hemorrhage
TBI	traumatic brain injury
